# *N*-(1-Acetyl-5-benzoyl-1,4,5,6-tetra­hydro­pyrrolo­[3,4-*c*]pyrazol-3-yl)benzamide

**DOI:** 10.1107/S1600536812019708

**Published:** 2012-05-12

**Authors:** Xiao-Guang Bai, Ju-Xian Wang, Yu-Cheng Wang

**Affiliations:** a Institute of Medicinal Biotechnology, Chinese Academy of Medical Sciences and Peking Union Medical College, Beijing 100050, People’s Republic of China

## Abstract

In the mol­ecule of the title compound, C_21_H_18_N_4_O_3_, the fused pyrrolo­[3,4-*c*]pyrazole ring system is approximately planar [maximum deviation = 0.0486 (16) Å] and forms dihedral angles of 87.21 (8) and 35.46 (7)° with the phenyl rings. In the crystal, N—H⋯O and C—H⋯O hydrogen bonds and weak C—H⋯π inter­actions link the mol­ecules into chains parallel to [201].

## Related literature

For background to potential anti­cancer kinase inhibitors, see: Fancelli *et al.* (2005 ▶); Gadekar *et al.* (1968 ▶). For the structures of related compounds synthesized by our group, see: Guo *et al.* (2010 ▶); Xia *et al.* (2011 ▶).
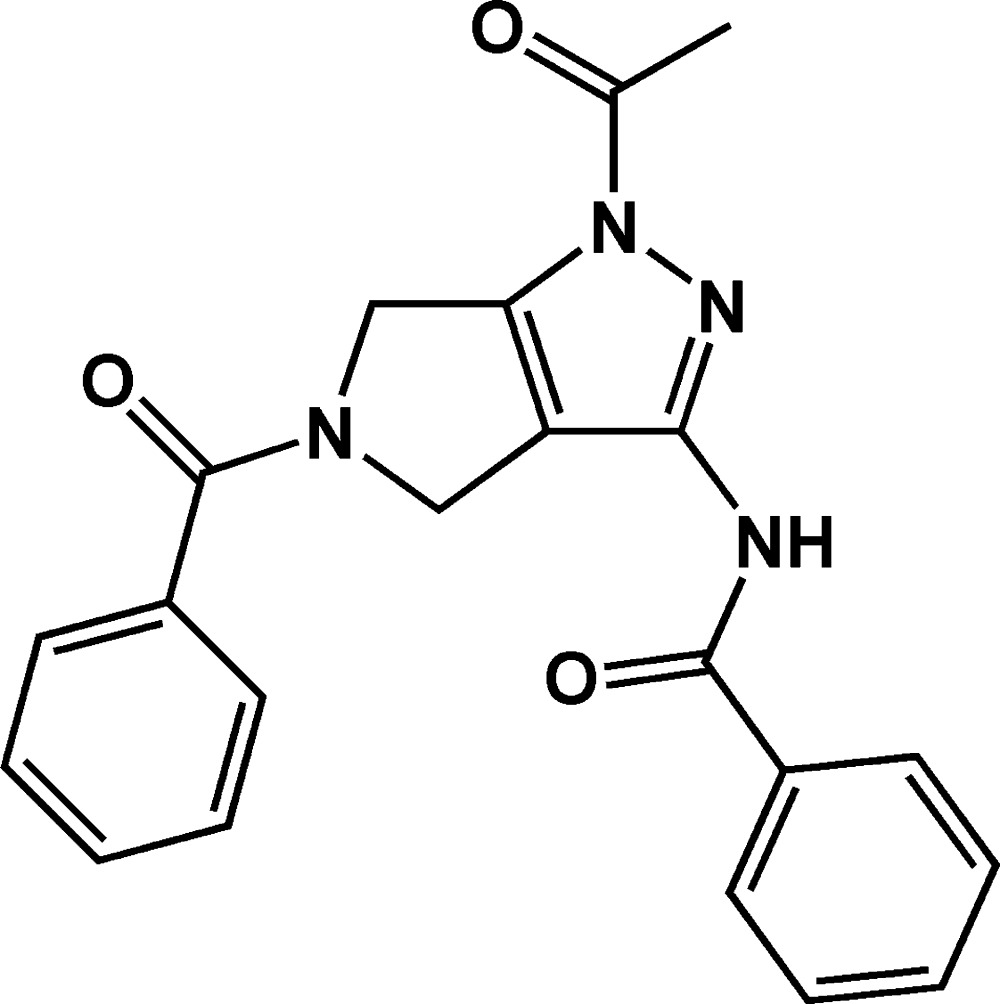


## Experimental

### 

#### Crystal data

C_21_H_18_N_4_O_3_
*M* *_r_* = 374.39
Monoclinic, 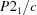

*a* = 5.32163 (11) Å
*b* = 21.1878 (5) Å
*c* = 16.4585 (3) Å
β = 96.9378 (17)°
*V* = 1842.16 (7) Å^3^
*Z* = 4
Cu *K*α radiation
μ = 0.76 mm^−1^
*T* = 293 K
0.25 × 0.22 × 0.18 mm

#### Data collection

Oxford Diffraction Xcalibur Atlas Gemini ultra diffractometer
Absorption correction: multi-scan (*CrysAlis PRO*; Agilent, 2011 ▶) *T*_min_ = 0.819, *T*_max_ = 1.000
10256 measured reflections
3276 independent reflections
2856 reflections with *I* > 2σ(*I*)
*R*_int_ = 0.018

#### Refinement

*R*[*F* ^2^ > 2σ(*F* ^2^)] = 0.053
*wR*(*F* ^2^) = 0.150
*S* = 1.07
3276 reflections
254 parameters
H-atom parameters constrained
Δρ_max_ = 0.62 e Å^−3^
Δρ_min_ = −0.25 e Å^−3^

### 

Data collection: *CrysAlis PRO* (Agilent, 2011 ▶); cell refinement: *CrysAlis PRO*; data reduction: *CrysAlis PRO*; program(s) used to solve structure: *SHELXS97* (Sheldrick, 2008 ▶); program(s) used to refine structure: *SHELXL97* (Sheldrick, 2008 ▶); molecular graphics: *SHELXTL* (Sheldrick, 2008 ▶); software used to prepare material for publication: *SHELXTL*.

## Supplementary Material

Crystal structure: contains datablock(s) I, global. DOI: 10.1107/S1600536812019708/rz2745sup1.cif

Structure factors: contains datablock(s) I. DOI: 10.1107/S1600536812019708/rz2745Isup2.hkl

Supplementary material file. DOI: 10.1107/S1600536812019708/rz2745Isup3.cml

Additional supplementary materials:  crystallographic information; 3D view; checkCIF report

## Figures and Tables

**Table 1:** Hydrogen-bond geometry (Å, °) *Cg*1 is the centroid of the C16–C21 phenyl ring.

*D*—H⋯*A*	*D*—H	H⋯*A*	*D*⋯*A*	*D*—H⋯*A*
N4—H4⋯O1^i^	0.86	2.23	2.997 (2)	148
C20—H20⋯O1^ii^	0.93	2.49	3.359 (3)	156
C5—H5*A*⋯*Cg*1^ii^	0.97	2.64	3.508 (3)	150

Symmetry codes: (i) 
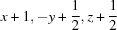
; (ii) 
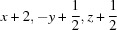
.

**Fig. 1.**
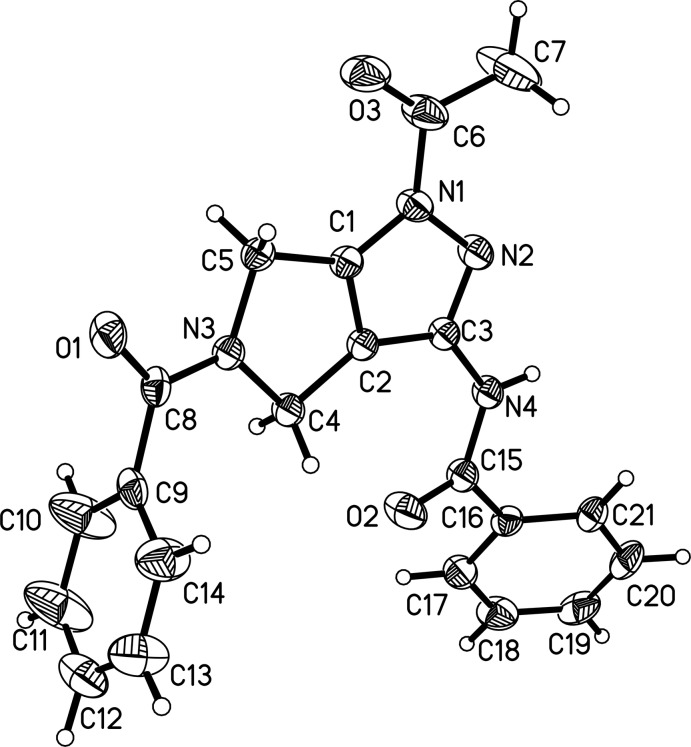
The molecular structure of the title compound, with displacement ellipsoids are drawn at the 30% probability level.

**Fig. 2.**
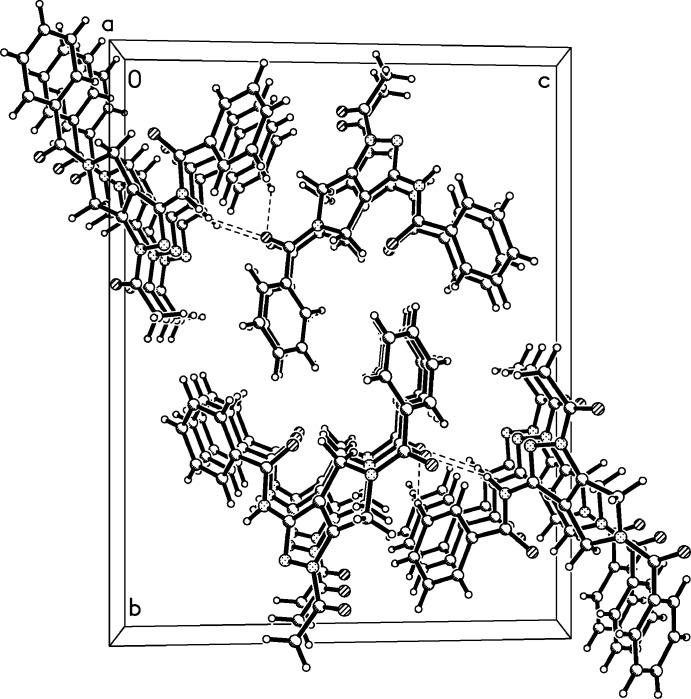
Packing diagram of the title compound viewed along the a axis. Hydrogen bonds are shown as dashed lines.

## supplementary crystallographic information

### Comment

In our ongoing project (Guo *et al.*, 2010; Xia *et al.*, 2011) devoted to the development of potential anticancer kinase inhibitors (Fancelli *et al.*, 2005; Gadekar *et al.*, 1968), we have synthesized the title compound and report its crystal structure herein.

In the molecule of the title compound (Fig. 1), bond lengths and angles have normal values. The fused pyrrole-pyrazole ring system is approximately planar (maximum deviation 0.0486 (16) Å for atom N3), the dihedral angle between the two five-membered rings being 1.32 (14)°. The phenyl rings C9–C14 and C16–C21 form dihedral angles of 87.21 (8) and 35.46 (7)°, respectively, with the mean plane through C1/N1/N2/C3/C2/C4/N3/C5. In the crystal structure (Fig. 2), molecules are linked by intermolecular N—H···O and C—H···O hydrogen bonds (Table 1) and by weak C—H···π interactions to form chains running parallel to the [2 0 1] direction.

### Experimental

A solution of benzoyl chloride (3.37 g, 24 mmol) in THF (20 ml) was added slowly to a mixture of 5-*tert*-butyl 1-ethyl 3-aminopyrrolo[3,4-*c*]pyrazole-1,5(4*H*,6H)-dicarboxylate (6.5 g, 21.8 mmol) and DIEA (N,N-diisopropylethylamine; 24 ml, 130.8 mmol) in THF (250 ml) at 0–5 °C for 12 h, the resulting suspension was evaporated under vacuum to dryness, and the residual was taken up with AcOEt and water, the organic layer was separated and stayed for 2 h to form white solid in the solution, separated by filtration and washed with Et2O, to give 7.37 g (84.5%) of 5-*tert*-butyl 1-ethyl 3-benzamidopyrrolo[3,4-*c*]pyrazole-1,5(4*H*,6H)-dicarboxylate as a white solid. A suspension of 5-*tert*-butyl 1-ethyl 3-benzamidopyrrolo[3,4-*c*]pyrazole-1,5(4*H*,6H)-dicarboxylate (7.6 g, 19 mmol) in DCM (300 ml) was pumped dried hydrochloride gas under room temperature for 3 h, filtered, extensively washed with Et2O, and dried under vacuum at 40 °C to give 6.37 g (100%) of ethyl 3-benzamido-5,6-dihydropyrrolo[3,4-*c*]pyrazole-1(4*H*)-carboxylate hydrochloride as white powder. A solution of benzoyl chloride (3.04 g, 21.6 mmol) in THF (20 ml) was added slowly to a suspension of ethyl 3-benzamido-5,6-dihydropyrrolo[3,4-*c*]pyrazole-1(4*H*)-carboxylate hydrochloride (6.05 g, 18 mmol) and DIEA (17.8 ml, 108 mmol) in THF (200 ml) at 0–5 °C, The resulting suspension was evaporated under vacuum to dryness, and the residual was taken up with AcOEt and water, the organic layer was separated and stayed for 2 h to form white solid in the solution, separated by filtration and washed with Et2O, to give 7.1 g (97.6%) of ethyl 3-benzamido-5-benzoyl-5,6-dihydropyrrolo[3,4-*c*]pyrazole-1(4*H*)-carboxylate as a white solid. A solution of ethyl 3-benzamido-5-benzoyl-5,6-dihydropyrrolo[3,4-*c*]pyrazole-1(4*H*)-carboxylate (6.8 g, 16.8 mmol) in MeOH (300 ml) and Et3N (30 ml) was stirred at room temperature for 2 h. The resulting mixture was evaporated to dryness and dried under vacuum to give 5.47 g (98%) of *N*-(5-benzoyl-1,4,5,6-tetrahydropyrrolo[3,4-*c*]pyrazol-3-yl)benzamide as white powder. A solution of acetyl chloride (0.424 g, 5.4 mmol) in THF (10 ml) was added slowly to a mixture of *N*-(5-benzoyl-1,4,5,6-tetrahydropyrrolo[3,4-*c*]pyrazol-3-yl)benzamide (1.5 g, 4.5 mmol) and DIEA (4.5 ml, 27 mmol) in THF (80 ml) at 0–5 °C for 12 h, the resulting suspension was evaporated under vacuum to dryness, and the residual was taken up with AcOEt and water, the organic layer was separated and stayed for 2 h to form white solid in the solution, separated by filtration and washed with Et2O, to give 1.5 g (89%) of *N*-(1-acetyl-5-benzoyl-1,4,5,6-tetrahydropyrrolo[3,4-*c*]pyrazol-3-yl)benzamide as a white solid. Colourless block crystals suitable for X-ray diffraction were obtained in 6 days by slow envaporation of a mixed solution (1:1 *v*/*v*) of dichloromethane and ethyl acetate.

### Refinement

All H atoms were placed in calculated positions and refined using a riding motion approximation, with C—H = 0.93–0.97 Å, N—H=0.86 Å, and with *U*_iso_(H) = 1.2*U*_eq_(C, N) or 1.5*U*_eq_(C) for methyl H atoms.

### Crystal data

**Table:**

C_21_H_18_N_4_O_3_	*F*(000) = 784
*M**_r_* = 374.39	*D*_x_ = 1.350 Mg m^−^^3^
Monoclinic, *P*2_1_/*c*	Cu *K*α radiation, λ = 1.54184 Å
Hall symbol: -P 2ybc	Cell parameters from 5825 reflections
*a* = 5.32163 (11) Å	θ = 3.4–66.8°
*b* = 21.1878 (5) Å	µ = 0.76 mm^−^^1^
*c* = 16.4585 (3) Å	*T* = 293 K
β = 96.9378 (17)°	Block, colorless
*V* = 1842.16 (7) Å^3^	0.25 × 0.22 × 0.18 mm
*Z* = 4	

### Data collection

**Table:**

Oxford Diffraction Xcalibur Atlas Gemini ultra diffractometer	3276 independent reflections
Radiation source: Enhance Ultra (Cu) X-ray Source	2856 reflections with *I* > 2σ(*I*)
Mirror monochromator	*R*_int_ = 0.018
Detector resolution: 10.4713 pixels mm^-1^	θ_max_ = 66.9°, θ_min_ = 3.4°
ω scans	*h* = −6→5
Absorption correction: multi-scan (*CrysAlis PRO*; Agilent, 2011)	*k* = −25→23
*T*_min_ = 0.819, *T*_max_ = 1.000	*l* = −19→18
10256 measured reflections	

### Refinement

**Table:**

Refinement on *F*^2^	Primary atom site location: structure-invariant direct methods
Least-squares matrix: full	Secondary atom site location: difference Fourier map
*R*[*F*^2^ > 2σ(*F*^2^)] = 0.053	Hydrogen site location: inferred from neighbouring sites
*wR*(*F*^2^) = 0.150	H-atom parameters constrained
*S* = 1.07	*w* = 1/[σ^2^(*F*_o_^2^) + (0.0752*P*)^2^ + 0.8604*P*] where *P* = (*F*_o_^2^ + 2*F*_c_^2^)/3
3276 reflections	(Δ/σ)_max_ < 0.001
254 parameters	Δρ_max_ = 0.62 e Å^−^^3^
0 restraints	Δρ_min_ = −0.25 e Å^−^^3^

### Special details

**Table:**

Geometry. All e.s.d.'s (except the e.s.d. in the dihedral angle between two l.s. planes) are estimated using the full covariance matrix. The cell e.s.d.'s are taken into account individually in the estimation of e.s.d.'s in distances, angles and torsion angles; correlations between e.s.d.'s in cell parameters are only used when they are defined by crystal symmetry. An approximate (isotropic) treatment of cell e.s.d.'s is used for estimating e.s.d.'s involving l.s. planes.
Refinement. Refinement of *F*^2^ against ALL reflections. The weighted *R*-factor *wR* and goodness of fit *S* are based on *F*^2^, conventional *R*-factors *R* are based on *F*, with *F* set to zero for negative *F*^2^. The threshold expression of *F*^2^ > σ(*F*^2^) is used only for calculating *R*-factors(gt) *etc*. and is not relevant to the choice of reflections for refinement. *R*-factors based on *F*^2^ are statistically about twice as large as those based on *F*, and *R*- factors based on ALL data will be even larger.

### Fractional atomic coordinates and isotropic or equivalent isotropic displacement parameters (Å^2^)

**Table:**

	*x*	*y*	*z*	*U*_iso_*/*U*_eq_	
O1	−0.5691 (3)	0.31747 (7)	0.32789 (9)	0.0590 (4)	
N4	0.3203 (3)	0.24185 (8)	0.66211 (9)	0.0422 (4)	
H4	0.3904	0.2161	0.6983	0.051*	
N3	−0.3129 (3)	0.29957 (8)	0.44421 (10)	0.0421 (4)	
N2	0.0354 (3)	0.16211 (8)	0.61696 (10)	0.0481 (4)	
N1	−0.1782 (3)	0.15647 (8)	0.56051 (10)	0.0459 (4)	
O2	0.3492 (4)	0.33753 (8)	0.60417 (11)	0.0722 (6)	
C8	−0.3981 (4)	0.33561 (9)	0.37999 (11)	0.0421 (4)	
C3	0.1126 (3)	0.22096 (9)	0.60959 (10)	0.0380 (4)	
C1	−0.2243 (4)	0.21181 (9)	0.51951 (11)	0.0387 (4)	
O3	−0.5009 (4)	0.10010 (8)	0.49746 (12)	0.0764 (6)	
C2	−0.0472 (3)	0.25406 (9)	0.54786 (10)	0.0370 (4)	
C5	−0.4179 (4)	0.23584 (9)	0.45428 (11)	0.0413 (4)	
H5A	−0.4240	0.2111	0.4045	0.050*	
H5B	−0.5850	0.2375	0.4721	0.050*	
C4	−0.0927 (4)	0.31603 (9)	0.50527 (12)	0.0449 (5)	
H4A	−0.1351	0.3488	0.5425	0.054*	
H4B	0.0522	0.3292	0.4790	0.054*	
C15	0.4185 (4)	0.30113 (9)	0.65912 (12)	0.0446 (5)	
C21	0.8086 (4)	0.27803 (11)	0.75721 (12)	0.0471 (5)	
H21	0.8090	0.2366	0.7384	0.057*	
C16	0.6190 (4)	0.31905 (9)	0.72611 (11)	0.0425 (4)	
C9	−0.2769 (4)	0.39859 (9)	0.37409 (12)	0.0456 (5)	
C17	0.6176 (4)	0.38031 (11)	0.75652 (14)	0.0562 (6)	
H17	0.4912	0.4083	0.7359	0.067*	
C20	0.9981 (4)	0.29859 (13)	0.81639 (14)	0.0600 (6)	
H20	1.1283	0.2713	0.8360	0.072*	
C6	−0.3162 (5)	0.10024 (11)	0.54676 (15)	0.0661 (7)	
C18	0.8033 (5)	0.39953 (12)	0.81712 (16)	0.0663 (7)	
H18	0.7987	0.4401	0.8384	0.080*	
C19	0.9951 (4)	0.35908 (14)	0.84631 (15)	0.0658 (7)	
H19	1.1225	0.3726	0.8862	0.079*	
C12	−0.0648 (6)	0.51684 (12)	0.35967 (17)	0.0757 (8)	
H12	0.0022	0.5568	0.3531	0.091*	
C14	−0.0915 (6)	0.40756 (13)	0.3251 (2)	0.0800 (8)	
H14	−0.0371	0.3738	0.2955	0.096*	
C10	−0.3456 (8)	0.44917 (14)	0.4169 (2)	0.1090 (14)	
H10	−0.4667	0.4440	0.4526	0.131*	
C7	−0.2147 (10)	0.04482 (15)	0.5952 (3)	0.144 (2)	
H7A	−0.0354	0.0418	0.5933	0.216*	
H7B	−0.2955	0.0071	0.5727	0.216*	
H7C	−0.2473	0.0496	0.6510	0.216*	
C11	−0.2405 (10)	0.50798 (14)	0.4088 (3)	0.1191 (16)	
H11	−0.2940	0.5418	0.4383	0.143*	
C13	0.0164 (7)	0.46637 (15)	0.3188 (2)	0.0912 (10)	
H13	0.1462	0.4716	0.2863	0.109*	

### Atomic displacement parameters (Å^2^)

**Table:**

	*U*^11^	*U*^22^	*U*^33^	*U*^12^	*U*^13^	*U*^23^
O1	0.0642 (10)	0.0544 (9)	0.0505 (8)	0.0047 (7)	−0.0257 (7)	−0.0032 (7)
N4	0.0440 (9)	0.0414 (9)	0.0372 (8)	−0.0003 (7)	−0.0114 (7)	0.0038 (6)
N3	0.0397 (8)	0.0425 (9)	0.0404 (8)	−0.0025 (6)	−0.0106 (7)	0.0033 (7)
N2	0.0570 (10)	0.0412 (9)	0.0413 (9)	−0.0033 (7)	−0.0135 (7)	0.0027 (7)
N1	0.0542 (10)	0.0384 (9)	0.0410 (9)	−0.0075 (7)	−0.0103 (7)	0.0016 (7)
O2	0.0808 (12)	0.0558 (10)	0.0684 (11)	−0.0185 (8)	−0.0383 (9)	0.0207 (8)
C8	0.0409 (10)	0.0441 (10)	0.0385 (9)	0.0099 (8)	−0.0066 (8)	−0.0028 (8)
C3	0.0415 (10)	0.0392 (10)	0.0312 (9)	0.0001 (8)	−0.0037 (7)	−0.0005 (7)
C1	0.0408 (10)	0.0405 (10)	0.0332 (9)	−0.0033 (8)	−0.0025 (7)	−0.0009 (7)
O3	0.0830 (12)	0.0615 (11)	0.0758 (11)	−0.0270 (9)	−0.0268 (10)	0.0046 (9)
C2	0.0382 (9)	0.0390 (9)	0.0320 (9)	−0.0013 (7)	−0.0032 (7)	0.0001 (7)
C5	0.0388 (10)	0.0436 (10)	0.0393 (10)	−0.0039 (8)	−0.0042 (8)	−0.0007 (8)
C4	0.0438 (10)	0.0424 (10)	0.0439 (10)	−0.0044 (8)	−0.0140 (8)	0.0051 (8)
C15	0.0432 (11)	0.0447 (11)	0.0424 (10)	−0.0015 (8)	−0.0097 (8)	0.0029 (8)
C21	0.0381 (10)	0.0599 (12)	0.0422 (10)	0.0033 (9)	0.0002 (8)	0.0017 (9)
C16	0.0373 (10)	0.0487 (11)	0.0393 (10)	−0.0054 (8)	−0.0039 (8)	0.0027 (8)
C9	0.0526 (11)	0.0414 (10)	0.0387 (10)	0.0096 (8)	−0.0109 (8)	0.0033 (8)
C17	0.0543 (12)	0.0482 (12)	0.0616 (13)	−0.0054 (10)	−0.0119 (10)	0.0011 (10)
C20	0.0346 (11)	0.0916 (18)	0.0508 (12)	0.0024 (11)	−0.0067 (9)	0.0084 (12)
C6	0.0853 (17)	0.0459 (13)	0.0605 (14)	−0.0183 (12)	−0.0185 (13)	0.0035 (10)
C18	0.0727 (16)	0.0577 (14)	0.0636 (14)	−0.0207 (12)	−0.0114 (12)	−0.0061 (11)
C19	0.0480 (13)	0.0912 (19)	0.0534 (13)	−0.0250 (12)	−0.0130 (10)	0.0008 (12)
C12	0.109 (2)	0.0471 (14)	0.0671 (16)	−0.0103 (14)	−0.0054 (15)	0.0079 (12)
C14	0.0849 (19)	0.0602 (16)	0.100 (2)	−0.0115 (13)	0.0312 (17)	−0.0197 (14)
C10	0.171 (4)	0.0469 (15)	0.127 (3)	−0.0042 (18)	0.093 (3)	−0.0123 (16)
C7	0.198 (5)	0.0529 (18)	0.153 (4)	−0.045 (2)	−0.095 (3)	0.036 (2)
C11	0.201 (4)	0.0424 (15)	0.130 (3)	−0.001 (2)	0.085 (3)	−0.0123 (17)
C13	0.101 (2)	0.076 (2)	0.102 (2)	−0.0246 (17)	0.0325 (19)	−0.0060 (17)

### Geometric parameters (Å, º)

**Table:**

O1—C8	1.234 (2)	C21—H21	0.9300
N4—C15	1.364 (3)	C16—C17	1.391 (3)
N4—C3	1.391 (2)	C9—C10	1.357 (3)
N4—H4	0.8600	C9—C14	1.361 (4)
N3—C8	1.338 (2)	C17—C18	1.378 (3)
N3—C5	1.478 (2)	C17—H17	0.9300
N3—C4	1.490 (2)	C20—C19	1.374 (4)
N2—C3	1.323 (2)	C20—H20	0.9300
N2—N1	1.384 (2)	C6—C7	1.484 (4)
N1—C1	1.360 (2)	C18—C19	1.374 (4)
N1—C6	1.403 (3)	C18—H18	0.9300
O2—C15	1.212 (2)	C19—H19	0.9300
C8—C9	1.490 (3)	C12—C11	1.322 (5)
C3—C2	1.428 (2)	C12—C13	1.361 (4)
C1—C2	1.342 (3)	C12—H12	0.9300
C1—C5	1.485 (2)	C14—C13	1.381 (4)
O3—C6	1.197 (3)	C14—H14	0.9300
C2—C4	1.494 (3)	C10—C11	1.379 (5)
C5—H5A	0.9700	C10—H10	0.9300
C5—H5B	0.9700	C7—H7A	0.9600
C4—H4A	0.9700	C7—H7B	0.9600
C4—H4B	0.9700	C7—H7C	0.9600
C15—C16	1.488 (3)	C11—H11	0.9300
C21—C16	1.382 (3)	C13—H13	0.9300
C21—C20	1.385 (3)		
			
C15—N4—C3	123.38 (15)	C21—C16—C15	122.68 (19)
C15—N4—H4	118.3	C17—C16—C15	118.04 (18)
C3—N4—H4	118.3	C10—C9—C14	117.2 (2)
C8—N3—C5	120.87 (15)	C10—C9—C8	121.8 (2)
C8—N3—C4	124.28 (16)	C14—C9—C8	120.9 (2)
C5—N3—C4	114.54 (14)	C18—C17—C16	120.1 (2)
C3—N2—N1	105.01 (15)	C18—C17—H17	119.9
C1—N1—N2	110.03 (15)	C16—C17—H17	119.9
C1—N1—C6	126.34 (17)	C19—C20—C21	120.4 (2)
N2—N1—C6	123.57 (17)	C19—C20—H20	119.8
O1—C8—N3	121.50 (19)	C21—C20—H20	119.8
O1—C8—C9	121.48 (17)	O3—C6—N1	118.8 (2)
N3—C8—C9	117.02 (16)	O3—C6—C7	125.3 (2)
N2—C3—N4	118.38 (16)	N1—C6—C7	115.8 (2)
N2—C3—C2	111.31 (16)	C19—C18—C17	120.4 (2)
N4—C3—C2	130.26 (17)	C19—C18—H18	119.8
C2—C1—N1	109.02 (16)	C17—C18—H18	119.8
C2—C1—C5	114.80 (17)	C20—C19—C18	119.8 (2)
N1—C1—C5	136.18 (17)	C20—C19—H19	120.1
C1—C2—C3	104.63 (16)	C18—C19—H19	120.1
C1—C2—C4	110.84 (16)	C11—C12—C13	118.8 (3)
C3—C2—C4	144.53 (17)	C11—C12—H12	120.6
N3—C5—C1	98.93 (14)	C13—C12—H12	120.6
N3—C5—H5A	112.0	C9—C14—C13	120.5 (3)
C1—C5—H5A	112.0	C9—C14—H14	119.7
N3—C5—H5B	112.0	C13—C14—H14	119.7
C1—C5—H5B	112.0	C9—C10—C11	121.7 (3)
H5A—C5—H5B	109.7	C9—C10—H10	119.1
N3—C4—C2	100.40 (15)	C11—C10—H10	119.1
N3—C4—H4A	111.7	C6—C7—H7A	109.5
C2—C4—H4A	111.7	C6—C7—H7B	109.5
N3—C4—H4B	111.7	H7A—C7—H7B	109.5
C2—C4—H4B	111.7	C6—C7—H7C	109.5
H4A—C4—H4B	109.5	H7A—C7—H7C	109.5
O2—C15—N4	121.98 (17)	H7B—C7—H7C	109.5
O2—C15—C16	121.14 (18)	C12—C11—C10	120.8 (3)
N4—C15—C16	116.88 (16)	C12—C11—H11	119.6
C16—C21—C20	120.0 (2)	C10—C11—H11	119.6
C16—C21—H21	120.0	C12—C13—C14	120.8 (3)
C20—C21—H21	120.0	C12—C13—H13	119.6
C21—C16—C17	119.25 (18)	C14—C13—H13	119.6
			
C3—N2—N1—C1	0.7 (2)	C3—N4—C15—O2	−8.7 (3)
C3—N2—N1—C6	178.1 (2)	C3—N4—C15—C16	172.23 (17)
C5—N3—C8—O1	1.1 (3)	C20—C21—C16—C17	−1.7 (3)
C4—N3—C8—O1	174.41 (19)	C20—C21—C16—C15	176.36 (19)
C5—N3—C8—C9	−178.70 (17)	O2—C15—C16—C21	−138.6 (2)
C4—N3—C8—C9	−5.4 (3)	N4—C15—C16—C21	40.5 (3)
N1—N2—C3—N4	176.94 (16)	O2—C15—C16—C17	39.5 (3)
N1—N2—C3—C2	−0.7 (2)	N4—C15—C16—C17	−141.4 (2)
C15—N4—C3—N2	178.88 (19)	O1—C8—C9—C10	98.7 (3)
C15—N4—C3—C2	−4.0 (3)	N3—C8—C9—C10	−81.5 (3)
N2—N1—C1—C2	−0.4 (2)	O1—C8—C9—C14	−81.3 (3)
C6—N1—C1—C2	−177.8 (2)	N3—C8—C9—C14	98.5 (3)
N2—N1—C1—C5	−179.4 (2)	C21—C16—C17—C18	−0.2 (3)
C6—N1—C1—C5	3.2 (4)	C15—C16—C17—C18	−178.4 (2)
N1—C1—C2—C3	0.0 (2)	C16—C21—C20—C19	1.9 (3)
C5—C1—C2—C3	179.24 (16)	C1—N1—C6—O3	−4.1 (4)
N1—C1—C2—C4	−179.16 (17)	N2—N1—C6—O3	178.9 (2)
C5—C1—C2—C4	0.1 (2)	C1—N1—C6—C7	175.1 (3)
N2—C3—C2—C1	0.5 (2)	N2—N1—C6—C7	−1.9 (4)
N4—C3—C2—C1	−176.80 (19)	C16—C17—C18—C19	1.9 (4)
N2—C3—C2—C4	179.1 (3)	C21—C20—C19—C18	−0.2 (4)
N4—C3—C2—C4	1.8 (4)	C17—C18—C19—C20	−1.7 (4)
C8—N3—C5—C1	167.03 (17)	C10—C9—C14—C13	−1.2 (5)
C4—N3—C5—C1	−6.9 (2)	C8—C9—C14—C13	178.8 (3)
C2—C1—C5—N3	4.0 (2)	C14—C9—C10—C11	2.7 (6)
N1—C1—C5—N3	−177.0 (2)	C8—C9—C10—C11	−177.3 (4)
C8—N3—C4—C2	−166.62 (18)	C13—C12—C11—C10	−1.9 (7)
C5—N3—C4—C2	7.0 (2)	C9—C10—C11—C12	−1.1 (7)
C1—C2—C4—N3	−4.1 (2)	C11—C12—C13—C14	3.3 (6)
C3—C2—C4—N3	177.3 (3)	C9—C14—C13—C12	−1.8 (5)

### Hydrogen-bond geometry (Å, º)

Cg1 is the centroid of the C16–C21 phenyl ring.

**Table:**

*D*—H···*A*	*D*—H	H···*A*	*D*···*A*	*D*—H···*A*
N4—H4···O1^i^	0.86	2.23	2.997 (2)	148
C20—H20···O1^ii^	0.93	2.49	3.359 (3)	156
C5—H5*A*···*Cg*1^ii^	0.97	2.64	3.508 (3)	150

Symmetry codes: (i) *x*+1, −*y*+1/2, *z*+1/2; (ii) *x*+2, −*y*+1/2, *z*+1/2.
